# Hyperprogressive disease after radiotherapy combined with anti-PD-1 therapy in renal cell carcinoma: a case report and review of the literature

**DOI:** 10.1186/s12894-021-00813-8

**Published:** 2021-03-21

**Authors:** Chao Liu, Jingjing Piao, Zhiyang Shang

**Affiliations:** 1 Department of Oncology, Zhuozhou Hospital, No.129, Fanyang Road, Zhuozhou City, Hebei Province China; 2 College of Nursing, Hebei University of Chinese Medicine, Shijiazhuang, Hebei Province China

**Keywords:** Hyperprogressive disease, Radiotherapy, Immunotherapy, Renal clear cell carcinoma, Case report

## Abstract

**Background:**

Studies have shown that immune checkpoint inhibitors (ICIs) have limited efficacy and can even increase tumour burden in short time periods. This is usually called hyperprogressive disease (HPD). To date, there are few reports regarding HPD; fewer have analysed the relationship between HPD and radiotherapy combined with ICIs, and their conclusions are controversial.

**Case presentation:**

A 42-year-old woman was diagnosed with stage IV renal clear cell carcinoma. The patient had previously received sorafenib and pazopanib as first- and second-line therapies, respectively. She received radiotherapy combined with nivolumab. Eighteen days after administration of the third dose of nivolumab, the patient’s general condition deteriorated; this was associated with immune-related adverse events. Computed tomography showed that the diameter of left lung metastases had sharply increased. A biopsy of the lung metastasis showed no infiltration of lymphocytes. The patient’s general condition worsened and she died of the disease on the 70th day after administration of the third dose of nivolumab.

**Conclusions:**

This report describes the development of HPD following the administration of radiotherapy combined with ICIs in a case of advanced renal cell carcinoma. The case indicates that radiotherapy may show bidirectional regulation effects on anti-tumour immune response. If the immunosuppressive function of radiotherapy is dominant, combined with ICIs, it could result in HPD.

**Supplementary Information:**

The online version contains supplementary material available at 10.1186/s12894-021-00813-8.

## Background

Immune checkpoint inhibitors (ICIs), especially antibodies against programmed death 1/programmed death-ligand 1 (PD-1/PD-L1) and cytotoxic T lymphocyte antigen-4 (CTLA-4), represent one of the most significant breakthroughs in the treatment of advanced malignancies; they have been approved to treat several solid tumours. However, despite the usefulness of these drugs, a significant number of patients do not respond to them. Therefore, improving the effectiveness of immunotherapy is a major goal of clinical research. Researchers have found that radiotherapy can promote the presentation of tumour antigens and induce immune-mediated anti-tumour responses, indicating that the combination of radiotherapy and ICIs may enhance the anti-tumour immune response [1, 2]. A secondary analysis of the KEYNOTE-001 phase 1 trial found that the overall survival (OS) was significantly longer in patients who received pembrolizumab and radiotherapy than in patients who had not previously received radiotherapy [3]. Moreover, the PACIFIC study showed that compared with placebo, durvalumab significantly prolonged progression-free survival (PFS) and OS among patients with stage III unresectable non-small-cell lung cancer (NSCLC) who did not have disease progression after concurrent chemoradiotherapy [4].

Recently, the number of active clinical trials testing ICIs combined with radiotherapy has seen a dramatic increase [5], and some reports have found that locally applied radiotherapy can improve the curative effect of ICIs [6–8]. However, studies have also shown that ICI therapy has limited efficacy, and can even accelerate tumour growth rate and increase tumour burden in short time periods. This is usually called hyperprogressive disease (HPD) [9]. HPD generally defined as > twofold increase in progression pace, > 50% increase in tumor burden and time to treatment failure < 2 months [10]. The median OS was significantly shortened in patients with HPD compared to those without the disease.

Until now, very few studies have analysed the relationship between HPD and radiotherapy combined with ICIs. Here, we present the case of a patient with advanced renal cell carcinoma who developed HPD following the administration of radiotherapy combined with nivolumab, and we review the literature on HPD associated with this treatment modality. This article hopes to provide new perspectives for researchers to explore the mechanisms underlying HPD.

## Case presentation

**Fig.1:**
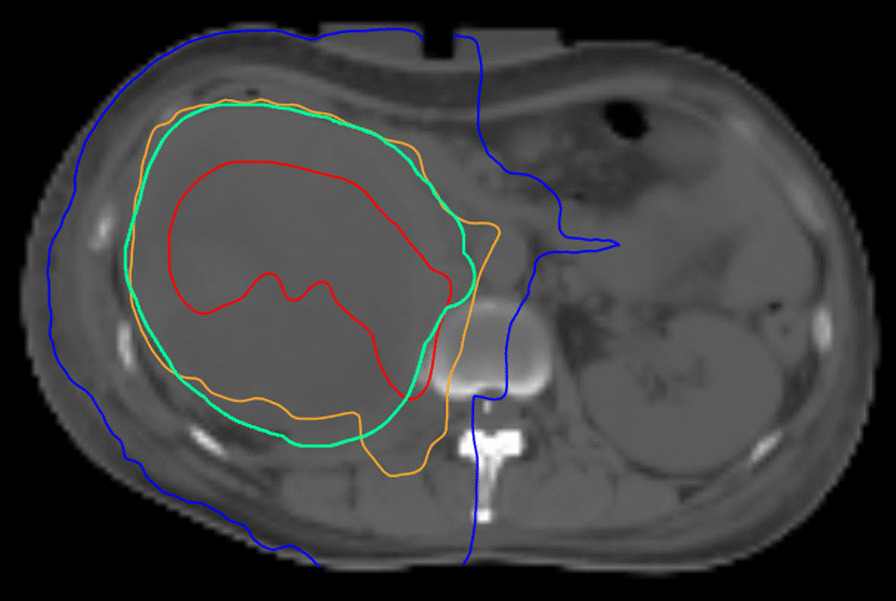
The treatment plan isodose distribution for right kidney lesion. Red indicates the planning target volume of hypofraction, and green indicates the planning target volume of conventional fraction

A 42-year-old woman was admitted to our hospital in March 2019 with a diagnosis of stage IV renal clear cell carcinoma (with multiple lung metastases). Her Eastern Cooperative Oncology Group (ECOG) and Royal Marsden Hospital (RMH) prognostic scores were 2 and 1 (albumin < 3.5 g/dL), respectively. The patient had previously received sorafenib and pazopanib as first- and second-line therapies, respectively, but these could not stop the progress of the disease. She had no history of autoimmune diseases, and her whole exome sequencing showed that the tumour mutation burden (TMB) was 127 mu/mb and no MDM2/4 amplification. The patient then received radiotherapy combined with nivolumab (180 mg, 3 mg/kg). The first dose of nivolumab was administered on 27 March 2019. After six days, she underwent radiotherapy. To induce systemic immune response and improve local tumour control, a suitable radiotherapy strategy was designed, including stereotactic body radiation therapy (SBRT) (24 Gy in 3 fractions) followed by the conventional 59.5 Gy in 17 fractions to the right kidney lesion (Fig. 1).

**Fig.2:**
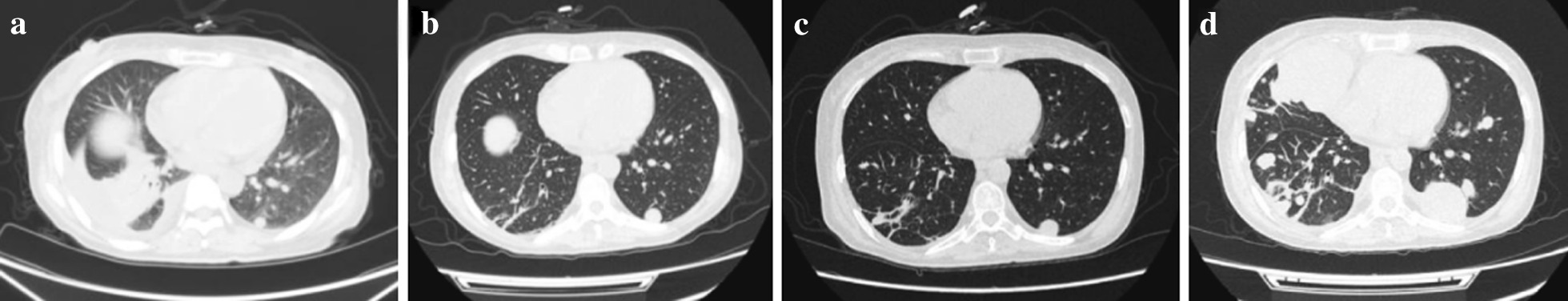
Chest CT scan performed before administration of the first dose of nivolumab (**a**), during radiotherapy (**b**), after radiotherapy (**c**), after administration of the third dose of nivolumab (**d**)

**Fig.3:**
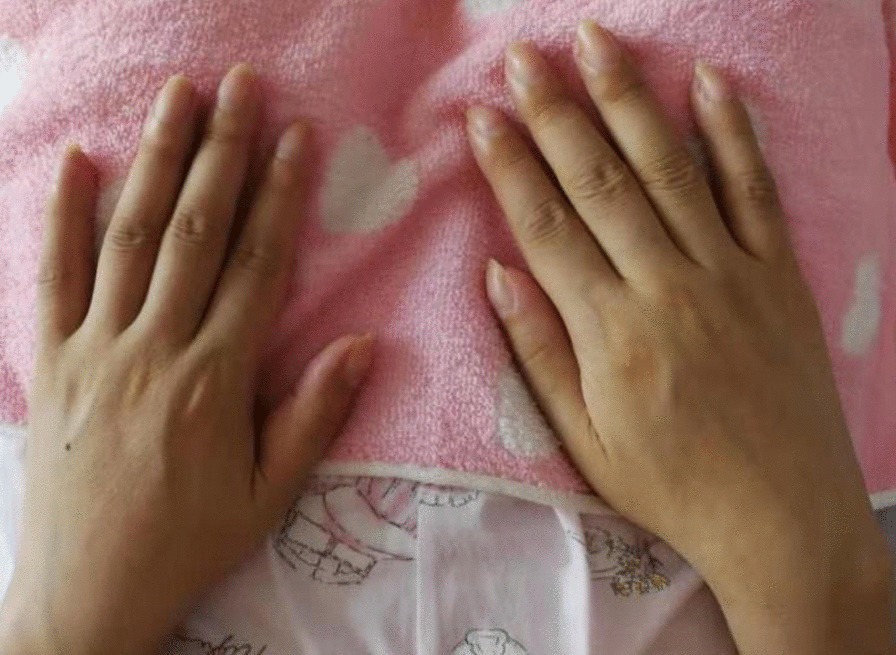
The patient's knuckles were deformed after administration of the third dose of nivolumab

**Fig.4:**
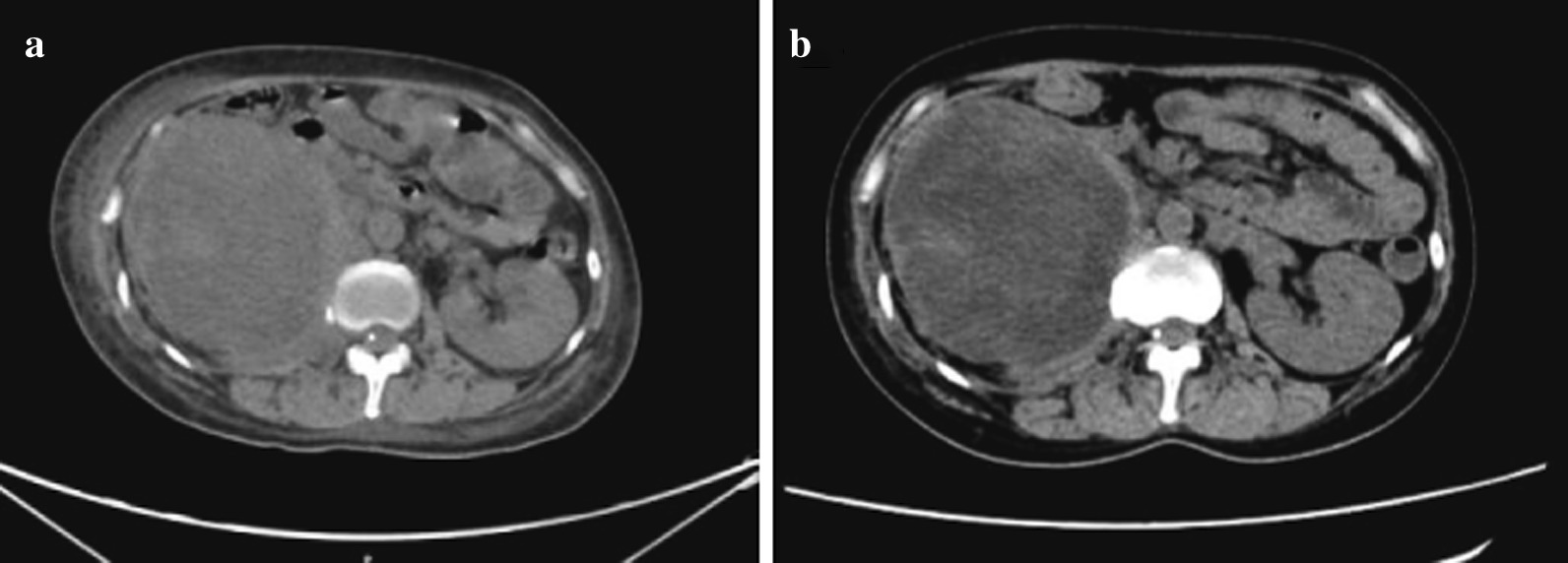
Abdominal CT scan performed before administration of the first dose of nivolumab (**a**), after administration of the third dose of nivolumab (**b**)

The diameter of the left lung metastases increased from 0.7 cm at baseline to 1.5 cm during radiotherapy, and showed no changes after completion of radiotherapy (Fig. 2a–c). After 14th days of radiotherapy, the patient was treated with the second and third doses of nivolumab (3 mg/kg every 2 weeks). Eighteen days after administration of the third dose of nivolumab, the patient’s general condition deteriorated; this was associated with an immune-related adverse event, namely, inflammatory arthritis, which presented as severe pain and deformed joints in the hands and knees (Fig. 3). Computed tomography scans of the right kidney lesions showed almost no change during treatment (Fig. 4a, b), but the diameter of the left lung metastases sharply increased to 4.9 (Fig. 2d). Moreover, the absolute count of peripheral lymphocytes decreased from 2.75 × 10^9^/L to 0.74 × 10^9^/L after radiotherapy.

**Fig.5:**
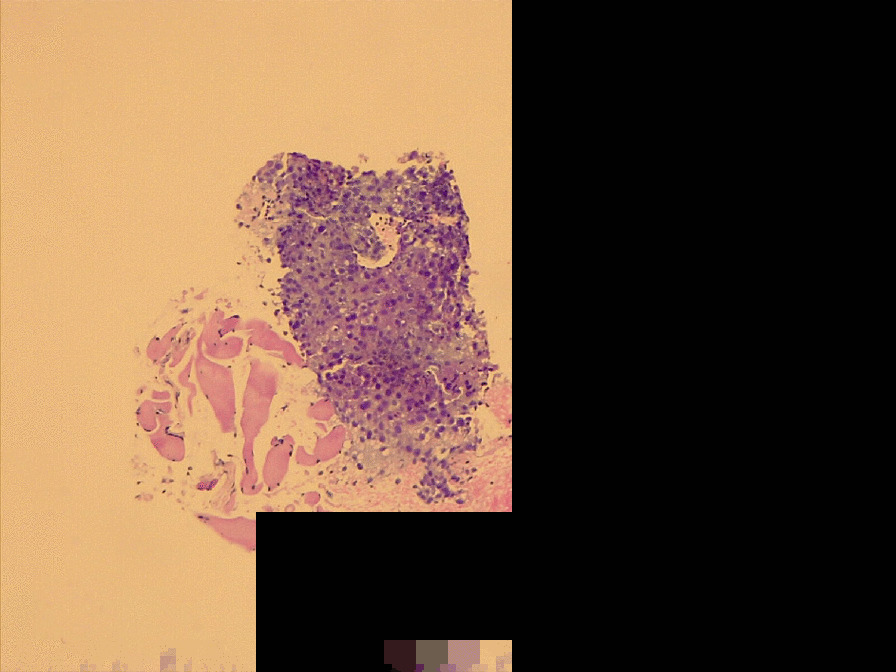
Haematoxylin and eosin staining of the lung metastasis biopsy sample showed no infiltration of lymphocytes

Radiological evaluation alone is not enough to distinguish pseudoprogression from HPD, so a biopsy of the lung metastasis was performed. It showed no infiltration of lymphocytes (Fig. 5 and Additional file 1: Fig. S1); thus, pseudoprogression was excluded. Subsequently, the patient received prednisone therapy. However, her general condition worsened and she received supportive care. She died of respiratory failure on the 70th day after administration of the third dose of nivolumab.

## Discussion and conclusions

It is hypothesized that the therapeutic effect of radiotherapy combined with ICIs should be better. However, in this case, radiotherapy combined with an ICI led to a significant increase in the size of the left lung metastasis, suggesting that radiotherapy induced HPD.

To date, there are few reports regarding HPD. In the literature, the incidence of HPD ranged from 1 to 7% for renal cell carcinoma and from 1 to 30% for other tumours [11]. Few studies have analysed the relationship between HPD and radiotherapy combined with ICIs, and their conclusions are controversial. Champiat et al. reported that ICI-related HPD occurred in twelve patients with malignant solid tumours. The authors found that HPD was not related to the type of previous treatment, including radiotherapy [9]. Ferrara et al. analysed the relevant factors in 56 advanced NSCLC patients with HPD and reached a similar conclusion [12]. However, in patients with recurrent and/or metastatic head and neck squamous cell carcinoma, the incidence of HPD was up to 29%. The authors of that study argued that radiotherapy might play a role since almost all cases of HPD occurred in patients who had at least one locoregional recurrence in an irradiated field [13]; however, they did not study the underlying mechanism. Nevertheless, their study reported the most information regarding the relationship between radiotherapy and HPD. To our knowledge, there are, in total, ten studies that involve radiotherapy and HPD (Table 1).

**Table 1:** Ten studies of HPD involved radiotherapy

Year	Author	Tumor types	The number of cases of HPD	Radiotherapy strategy	Type of ICB	Relationship between radiotherapy and HPD
2016	Champiat [9]	Solid tumors	12	Not described	Anti-PD-1 Anti-PD-L1	Unrelated
2016	Alexander [14]	Renal cancer	1	20 Gy in 5 fractions	Anti-PD-1	Unrelated
2017	Kato [15]	Endometrial stromal sarcoma	1	SBRT	Anti-PD-1	Not described
		Breast cancer	1	Not described	Anti-PD-1	Not described
2017	Saâda-Bouzid [13]	Head and neck cancers	10	Not described	Anti-PD-1 Anti-PD-L1	Related
2017	Yoshida T [16]	Lung cancer	1	60 Gy	Anti-PD-1	Related
2018	Ogata [17]	Gastric cancer	1	50.4 Gy in 28 fractions	Anti-PD-1	Related
2018	Du [18]	Lung cancer	1	8 Gy in 1 fraction	Anti-PD-1	Related
2018	Ferrara [12]	Lung cancer	56	Not described	Anti-PD-1 Anti-PD-L1	Not described
2019	Yilmaz [19]	Melanoma	1	Not described	Anti-PD-1	Related
2019	Bosch-Barrera [20]	Lung cancer	1	8 Gy in 1 fraction	Anti-PD-L1	Not described

Because radiotherapy is considered a double-edged sword in anti-tumour immunity, some investigators have suggested that HPD results from radiotherapy, which impairs the function of immune cells and changes the tumour microenvironment. Du et al. reported the case of a patient with NSCLC who developed HPD following SBRT combined with an ICI [18]. By analysing the biopsies of the irradiated target lesion after radiotherapy and prior to ICI treatment, they found that the absence of tumour-infiltrating lymphocytes (TILs) and cancer-intrinsic PD-1 expression may be the main causes of HPD. Further studies have revealed that blockade of cancer-intrinsic PD-1 can enhance cancer survival in vitro and in vivo. The authors proposed that radiotherapy prior to ICI treatment can increase type I interferon production by either tumour cells or immune cells, which may in turn increase cancer-intrinsic PD-1 expression; however, they did not analyse the biopsies before radiotherapy, so there is insufficient evidence to support this theory. Ogata et al. reported the case of a patient with advanced gastric cancer who received nivolumab seven days after the completion of radiotherapy, whereupon he experienced HPD within the irradiation field [17]. They suggested that radiotherapy alters the immune environment and facilitates the occurrence of HPD, but did not investigate this further.

Although the expression of cancer-intrinsic PD-1 was not detected in our patient, the lymphocyte counts sharply decreased after radiotherapy, and the absence of TILs may have been the cause of HPD. This is consistent with the report of Alexander et al [14]. Cho et al. also reported that lymphopenia was indicative of poor prognosis in NSCLC patients who received ICIs, and this was significantly associated with radiotherapy [21].

Regulatory T cells (Tregs), which function to suppress tumour-specific immunity, have been confirmed as radioresistant lymphocyte subpopulations in many studies [22–26]. Kamada et al. found that PD-1 blockade can markedly increase the number of Tregs in HPD patients and result in inhibition of anti-tumour immunity [27]. Romano et al. investigated 15 patients with stage IV cutaneous melanoma who responded to CTLA-4-specific monoclonal antibody, and found that anti-CTLA-4 therapy may enhance immune responses by target Tregs in vivo [28]. To our knowledge, there are no reports of patients who developed HPD following radiotherapy combined with anti-CTLA-4 therapy. Therefore, this combination may reduce the occurrence of HPD and improve the curative effect. Unfortunately, in our patient, biopsy of the lung metastasis showed no lymphocytes, and we did not detect the Treg fraction in the peripheral blood. Moreover, we were concerned about the side effects of anti-CTLA-4 therapy, and regardless, the patient would not have been able to receive anti-CTLA-4 therapy because this drug was not available in China.

The choice of radiotherapy dose is the other key aspect of the combination therapy, and SBRT may be the best option [29]. Compared with conventional fractionated radiotherapy, preclinical studies confirmed that SBRT can promote the host anti-tumour immune response through several potential mechanisms, including normalisation of microvessels to alleviate tumour hypoxia, improvement in efficient delivery of drugs, abundant neoantigen exposure, and recruitment of anti-tumour immune cells to alter the immunosuppressive tumour microenvironment [29–32]. Several clinical studies have reported that the combination of SBRT with immunotherapy has shown favourable curative effect [6, 33, 34]. Two recent clinical trials also found that ICIs combined with SBRT show good prospects for the treatment of renal cell carcinoma [35, 36]; therefore, our patient was treated with SBRT at a dose of 24 Gy for 3 fractions. However, there were also two cases reported in which the patients developed HPD following SBRT combined with ICIs. One NSCLC patient was mentioned above in this paper [18]. Another patient with endometrial stromal sarcoma experienced HPD after treatment with nivolumab combined with SBRT [15]; however, the strategy of combined treatment administered in this case was not described in detail. The patient was found to have MDM2 amplifications and that was considered the primary cause of HPD. Nevertheless, our patient did not have any gene mutation associated with HPD, including MDM2/MDM4 and EGFR.

While two cases are not enough to conclude that SBRT was associated with HPD, studies have also suggested that SBRT can suppress the immune system. For example, research has found that SBRT can increase the relative number of Tregs [23]. However, Cho et al. reported that compared with SBRT, multiple courses of radiotherapy and high total dose significantly increased the risk of lymphopenia in patients receiving immunotherapy for NSCLC, who showed poorer PFS and OS than those who did not receive immunotherapy [21]. In an attempt to activate the immune system and directly kill the local tumour, we administered SBRT followed by the conventional fractioned radiotherapy. This type of radiotherapy strategy may be counterproductive. Moreover, Tang et al. reported that a larger target volume was associated with severe lymphopenia [37]. These factors may have caused lymphopenia and HPD in our patient.

The patient had received sorafenib and pazopanib as first- and second-line treatments, respectively. However, Zhao et al. found that sorafenib may cause the loss of T-cell immune response by inducing apoptosis and targeting LCK [38]. Sunitinib, another renal cancer-targeted drug, has been found to prevent T-cell-mediated immune response [39]. Despite the aforementioned issues, ICIs combined with targeted drugs have already been approved for the treatment of advanced renal cell carcinoma, and there are no reports indicating a higher rate of HPD than that in other solid tumours to date. Therefore, targeted drugs may not be a risk factor for HPD.

The TMB of our patient was very high. Although the prognostic role of TMB in cancer patients receiving ICIs is controversial, some studies have found that high TMB predicts better efficacy of ICIs [40, 41]. However, Kim et al. reported that HPD is not associated with TMB and PD-L1 expression [42]. In addition, our patient developed severe immune-related adverse events. Several studies have found that immune-related adverse events are associated with a better outcome after treatment with ICIs [43–45]. However, Kanjanapan et al. reported that HPD was not associated with immune-related adverse events [46]. Therefore, the positive correlative factors of the effect of ICIs may be irrelevant to HPD.

In conclusion, this report describes the development of HPD in a case of advanced renal cell cancer following the administration of radiotherapy combined with an ICI. We argue that radiotherapy may show bidirectional regulation effects on anti-tumour immune response, and if the immunosuppressive function of these treatments is dominant, combination with ICIs could result in treatment failure or even HPD. In contrast, the positive correlative factors of the effect of ICI, including TMB and PD-L1 expression and immune-related adverse events, may be irrelevant to HPD. Further research is needed to elucidate the mechanisms of these associations.

## Supplementary Information

**Additional file 1: Fig. S1**. Haematoxylin and eosin staining of the lung metastasis biopsy sample showed no infiltration of lymphocytes.

## Data Availability

All data generated are included in this published article.

## Abbreviations

CTLA-4: Cytotoxic T lymphocyte antigen
HPD: Hyperprogressive disease
ICI: Immune checkpoint inhibitor
NSCLC: Non-small cell lung cancer
OS: Overall survival
PD-1: Programmed death 1
PD-L1: Programmed death-ligand 1
PFS: Progression-free survival
SBRT: Stereotactic body radiation therapy
TIL: Tumour-infiltrating lymphocyte
TMB: Tumour mutation burden
