# Supplementary figures and images for: Hyperprogressive disease after radiotherapy combined with anti-PD-1 therapy in renal cell carcinoma: a case report and review of the literature
Source: BMC Urol. 2021 Mar 21;21:42. doi: 10.1186/s12894-021-00813-8 (PMC7981866; doi:10.1186/s12894-021-00813-8)

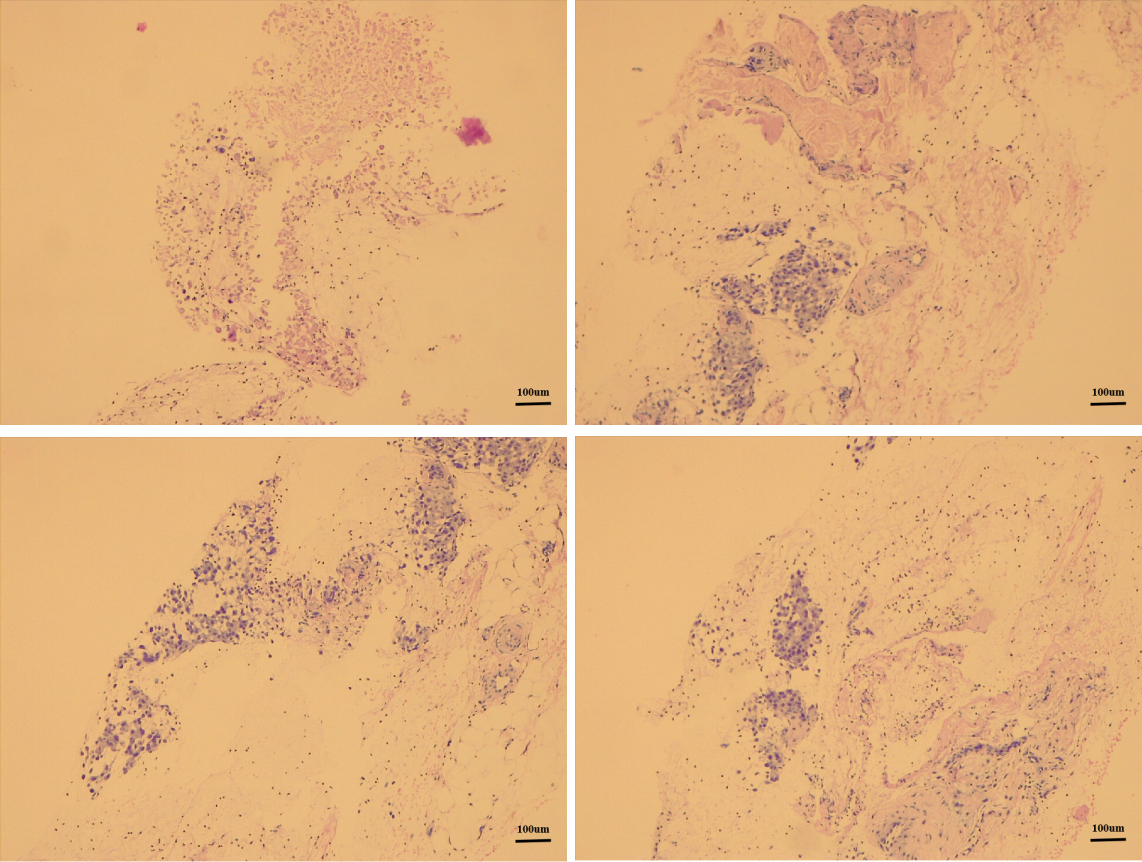

Supplement: Supplementary file 1 — Additional file 1: Fig. S1. Haematoxylin and eosin staining of the lung metastasis biopsy sample showed no infiltration of lymphocytes. [file 12894_2021_813_MOESM1_ESM.tif]
